# Discriminating between HuR and TTP binding sites using the *k*-spectrum kernel method

**DOI:** 10.1371/journal.pone.0174052

**Published:** 2017-03-23

**Authors:** Shweta Bhandare, Debra S. Goldberg, Robin Dowell

**Affiliations:** 1 Department of Computer Science, University of Colorado at Boulder, 1111 Engineering Dr, Boulder, CO, 80303 United States of America; 2 Computational Bioscience Program, University of Colorado, School of Medicine, 12801 E. 17th Ave., RC1N-6129, Aurora, CO, 80045, United States of America; 3 Department of Molecular, Cellular and Developmental Biology and the BioFrontiers Institute, University of Colorado at Boulder, 596 UCB, Boulder, Boulder, CO, 80309, United States of America; Medical Faculty Mannheim, University of Heidelberg, GERMANY

## Abstract

**Background:**

The RNA binding proteins (RBPs) human antigen R (HuR) and Tristetraprolin (TTP) are known to exhibit competitive binding but have opposing effects on the bound messenger RNA (mRNA). How cells discriminate between the two proteins is an interesting problem. Machine learning approaches, such as support vector machines (SVMs), may be useful in the identification of discriminative features. However, this method has yet to be applied to studies of RNA binding protein motifs.

**Results:**

Applying the *k*-spectrum kernel to a support vector machine (SVM), we first verified the published binding sites of both HuR and TTP. Additional feature engineering highlighted the U-rich binding preference of HuR and AU-rich binding preference for TTP. Domain adaptation along with multi-task learning was used to predict the common binding sites.

**Conclusion:**

The distinction between HuR and TTP binding appears to be subtle content features. HuR prefers strongly U-rich sequences whereas TTP prefers AU-rich as with increasing A content, the sequences are more likely to be bound only by TTP. Our model is consistent with competitive binding of the two proteins, particularly at intermediate AU-balanced sequences. This suggests that fine changes in the A/U balance within a untranslated region (UTR) can alter the binding and subsequent stability of the message. Both feature engineering and domain adaptation emphasized the extent to which these proteins recognize similar general sequence features. This work suggests that the *k*-spectrum kernel method could be useful when studying RNA binding proteins and domain adaptation techniques such as feature augmentation could be employed particularly when examining RBPs with similar binding preferences.

## Introduction

RNA binding proteins (RBPs) are crucial regulators of numerous post-transcriptional processes [1, 2]. RBPs identify their RNA targets in a highly specific fashion, through recognition of specific primary sequence and/or secondary structure. Many RNA binding proteins recognize AU-rich sequence elements, including human antigen R (HuR) and tristetraprolin (TTP). The association between AU-rich elements and particular proteins alters the stability of the RNA. For example, binding by HuR protects messenger RNA (mRNA) from degradation whereas binding of TTP promotes degradation. Recent high throughput photoactivatable-ribonucleoside-enhanced crosslinking and immunoprecipitation (PAR-CLIP) [3] data is available for these two key regulatory RNA binding proteins. This data suggests that both HuR and TTP bind to similar AU-rich elements which are typically 50–150 nucleotides long and generally located within the 3′ UTR. The extent to which sequence features discriminate between the two binding proteins remains an interesting question.

Previous efforts on understanding the AU-rich binding proteins HuR and TTP were focused primarily on identifying *k*-mer motifs. Mukherjee et al. [4] demonstrated that over 80% of the TTP sites in 3′UTRs overlap with HuR target binding sites. The motif recognized by HuR is more U-rich whereas the motif recognized by TTP is predominantly AU-rich [4]. However, it remains unclear to what extent these features discriminate between HuR and TTP binding *in vivo*. Position specific scoring matrix (PSSM)-based approaches are commonly utilized to describe protein binding [5–8]. These methods assume positional independence within the motif, which is sometimes also represented as a position frequency matrix (PFM). Another representation of motifs is *k*-mer based or consensus strings. This representation can capture intra-motif dependencies that are missed with the PSSM-based approach. Machine learning approaches that utilize *k*-mer searches factor in these dependencies [9–12], and in doing so can provide more discriminative power.

The *k*-spectrum kernel method has been used successfully in a number of bioinformatics applications. In protein sequence classification [13] it out-performed both BLAST [14] and Smith-Waterman [15] at super-family homology and fold recognition tasks. Beer et al. [16] demonstrated that the use of *k*-mer based (using DNA sequence elements as features) approach can be used to distinguish enhancers from random genomic regions as well as short transcription factor-binding sites for tissue specificity. Schultheiss et al. [17] successfully developed a Python pipeline called KIRMES using the spectrum kernel to identify degenerate motifs from micro-array data in Arabidopsis thaliana. Here we extend these applications to identify and characterize the distinct binding motifs of RNA binding proteins HuR and TTP.

## Materials and methods

### PAR-CLIP HuR and TTP clusters

Two papers have explored the binding targets of HuR and TTP. Lebedeva et al. [18] used PAR-CLIP [3] on unstressed HeLa cells to identify the binding targets of HuR. Reads were aligned and clusters of continuous read coverage were identified. Clusters were then filtered based on detectable nucleotide conversion events (a side effect of the crosslinking) and a quality score metric. The authors classified hits as **consensus** if detected in two out of three PAR-CLIP [3] experiments and **conservative** if detected in all three experiments. We utilized the conservative data-set for computational analysis such as identifying motifs. We obtained the data set as a BED file mapped to the hg19 genome from doRiNA [19]. Mukherjee et al. [4] carried out PAR-CLIP [3] experiments to identify the genome-wide binding sites for TTP. Reads were mapped to the genome and binding sites identified [20]. It is worth noting that Mukherjee directly compared their binding sites for TTP to previously published HuR data [21] using an consistent analysis pipeline. The final target list includes 4,626 peaks of mRNA-TTP interactions, downloaded from GEO (Gene Expression Omnibus) accession number GSE53185. For both data sets (positive set), peak statistics (minimum, average, and maximum length) are provided in Table 1. Additional information on the datasets for both RBPs is summarized in the Section HuR and TTP experimental methods of the S1 Appendix.

**Table 1:** Summary of sequence length of HuR and TTP PAR-CLIP [3] clusters.

	Number of Sequences	Max length	Min Length	Average Length
HuR	3642	243	19	56
TTP	4626	172	21	26

### Control sequences

For both the HuR and the TTP data-sets, a negative set of control sequences were generated using random 3′UTR transcripts from the human genome. For each binding sequence in the positive set, a size matched negative sequence was selected at a random location from a random transcript (not in the positive set). One control set was generated for each RNA binding protein.

### Computational tools for motif validation

To verify the motifs identified by Lebedeva et al. [18] and Mukherjee et al. [4], two primary sequence motif discovery tools were utilized. Discriminative regular expression motif elicitation (DREME) [22] is part of the MEME (multiple EM for motif elicitation) [23] tool suite (v 4.10.2) that uses a discriminative approach to motif discovery. DREME [22] was utilized with a motif length range of 7–13 nucleotides. DREME outputs the discriminatory *k*-mers along with E-value, p-value, and the position frequency matrix (PFM). The top five *k*-mers based on E-value were short-listed for comparison to the string kernel results [22]. The results list the over-represented *k*-mers (based on number of occurrences) associated with identified motifs. A second approach is the use of a string kernel function [24], a method that groups sequences with similar *k*-mers. This method has been used effectively to determine homology of protein sequences that share a remote evolutionary relationship [25]. A brief primer on SVMs and the *k*-spectrum kernel are provided in the Section k-spectrum Kernel Method of S1 Appendix. The *k*-spectrum kernel method was implemented using the PyML library (version PyML-0.7.13.3, Python version 2.7 [9] http://pyml.sourceforge.net).

For the *k*-spectrum kernel, the HuR, and TTP target sequences were each used independently to build *k*-spectrum kernel predictive models using a SVM. For each RNA binding protein (HuR and TTP), the model was built using the 80% of the sequences by iterating over three parameters; the SVM parameter C (cost of a mislabeling) and *k*-spectrum parameters K1 and K2 (*k*-mer length). The remaining 20% was held out as a validation set. The parameters *K*1 and *K*2 ranged between values 7 and 13 whereas the SVM parameter *C* ranged from 1e-10 to 10000 in powers of 10 increments. At each iteration (for a given set of parameters), the training set was run through a 10-fold stratified cross-validation. The receiver operating characteristic (ROC) score is used for optimization. By iterating through the parameters, the model optimized for ROC score is obtained, and is subsequently used to test unseen (validation set) data. The output of the model includes a list of feature *k*-mers and weights associated with each feature *k*-mer that was used to build the model. The feature *k*-mers are the support vectors that were used by the SVM to distinguish between the two classes. The value of the feature weight depicts the significance of the feature *k*-mer in the classification. The algorithm used to build the model is described in the Section Algorithm to build k-spectrum kernel models of S1 Appendix.

Results from the *k*-spectrum kernel method were evaluated based on multiple metrics: success rate (balanced success rate when the positive and negative classes are distinctly different sizes), sensitivity, positive predictive value (PPV) and area under the curve. To compute performance metrics from DREME, the PFM was utilized. In the positive set, scores greater than 0.6 * maximum score (threshold), were classified as true positive. Otherwise, the sequence is a false negative. For negative sequences, if the predicted motif score is greater than the threshold value, the sequence is classified as a false positive, and otherwise, it is a true negative. The score for every sequence is computed using TAMO [26], particularly the *MotifTools* python class.

### Discriminating between HuR and TTP binding sites using a common model

To dissect the features both common and specific to the motifs, the datasets (Section PAR-CLIP HuR and TTP clusters) were partitioned, using bedtools [27], into three subsets: **Data-set A** contained sequence clusters that bound HuR but not TTP (“HuR only”), **Data-set B** contained sequence clusters that bound TTP but not HuR (“TTP only”), and **Data-set C** contained clusters that were common to both HuR and TTP.

### Discriminating between HuR and TTP binding sites: Using multi-task learning and domain adaptation

**Fig 1:**
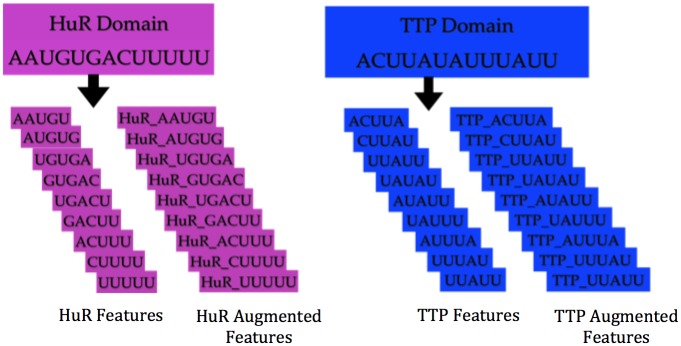
Feature Augmentation Technique. Original features and augmented features for HuR and TTP domains.

To identify the shared motifs between HuR and TTP, we utilized Multi-task Learning (MTL) as this approach leverages both the commonality and differences between the RBPs. To identify features specific and shared by domains, Daume III [28] proposed feature augmentation approach (a domain adaptation technique) wherein the feature space is tripled (when the number of domains = 2). For each feature in the original feature space, there is a “shared” version and two domain specific versions, which in our case are “HuR-specific” and “TTP-specific”. This is illustrated in Fig 1. The underlying algorithm is then expected to learn which features transfer across domains (hence shared features) and which do not (domain specific features). Domain adaptation selects features that are either domain specific (HuR or TTP specific) or shared in order to best explain the data. When a domain (HuR or TTP) specific feature is chosen, it implies that the sequence can be discriminated based on the domain specific knowledge, whereas when a shared feature is selected, the feature is shared between the domains. Each RBP constitutes a domain.

In order to run the *k*-spectrum kernel using domain adaptation, we constructed HuR (*M*_*H*_) and TTP (*M*_*T*_) models independently using 80% of the sequences for HuR and TTP. It is important to note that for this experiment, each model was generated from the original data-set; *M*_*H*_ was generated using all the HuR clusters, and similarly *M*_*T*_ from all the TTP clusters described in Section PAR-CLIP HuR and TTP clusters. The features identified by each model independently serve both as potential combined features and domain-specific features in the combined model. The domain specific features are prefixed with the RBP label (“HuR_” for features obtained from the HuR model, and “TTP_” for features obtained from the TTP model). The resulting combined model (*M*_*B*_) then identifies the most discriminative set of features that explain each domain from the combined features and domain specific sets.

## Results

### Predict RBP binding sites using the *k*-spectrum kernel

We first sought to determine whether the *k*-spectrum kernel could accurately and independently capture the known motifs without utilizing the cross-linking information. Prior work on identifying HuR motifs [4, 18, 29] utilized exclusively on the identification of 7-mers around the cross-linking site, identified by the alteration induced in the sequence. We utilized the *k*-spectrum kernel method on each RBP (HuR and TTP) independently leveraging only the cluster data which ignores the sequence change induced by the cross-linking. For both RBPs, we calculated the success rate of our *k*-spectrum model on held-out test data.

We first note that the *k*-mers used by the model to discriminate HuR clusters from the control data were consistent with the published HuR motifs (see Table 2). The HuR model classified the test data with a 77.3% success rate (see Table 2). Additionally, after U-rich *k*-mers the second highest scoring feature was AU-rich *k*-mers that correspond to the motifs identified by the miReduce [30] algorithm used by Lebedeva et al. [18]. Interestingly, the *k*-mers were generally longer (9 to 12 nucleotides) than the published 7-mers. Our results suggest that the published motif was flanked by U’s, consistent with the U-rich nature of HuR binding sites.

**Table 2:** Discriminative methods recover known K-mers for HuR. Top feature *k*-mers from the *k*-spectrum kernel and over-represented k-mers from DREME are compared to the published k-mers [18]. For the *k*-spectrum kernel, determined feature weights are provided.

Method	Success Rate	Sensitivity	PPV	ROC
*k*-spectrum	77.3	96.8	69.7	89.4
DREME	78.6	92.5	64.1	87.3
		**Published Motifs** [18]	***k*-spectrum Features and Weights**	**DREME over-represented *k*-mers**
		**U-rich**		
		UUUUUUU	UUUUUUUUU, 63.5	UUUUUUU
		UUUAUUU	UUUUAUUUU, 23.9	UUUUUUA
		UUUGUUU	UUUUUGUUU, 11.3	AUUUUUU
		**AU-rich**		
		UAUUUAU	UUUAUUUUU, 19.9	UUUUUUA
		AUUUUUA	AUUUUUUUU, 18.4	UUUUUAA
		AUUUAUU	UUUUUUUAA, 12.9	AUUUUUA
		AAUUUUUA	UUUUUAUUU, 10.5	AUUUAU
		AAUUUUA	AUUUUUAUUU, 7.49	AUUUAA
		AAUAUUU	AAUUUUUUU, 11.2	
		**Polypyrimidine Tract/Intronic**		
		CUUUUUUUU	CUUUUUUUU, 15.19	CUUUUUU
		UCUCUUUU	UUUCUUUUU, 13.04	UCUCUUUU
		UUUCUUU	UUUUCUUUU, 20.8	CUUUUUA
		UUUCCUU	UUUUUCUUU, 18.6	AUUUUCU
		UUUUUUUUC	UUUUUUUUC, 15.9	CUUUUAU

**Fig 2:**
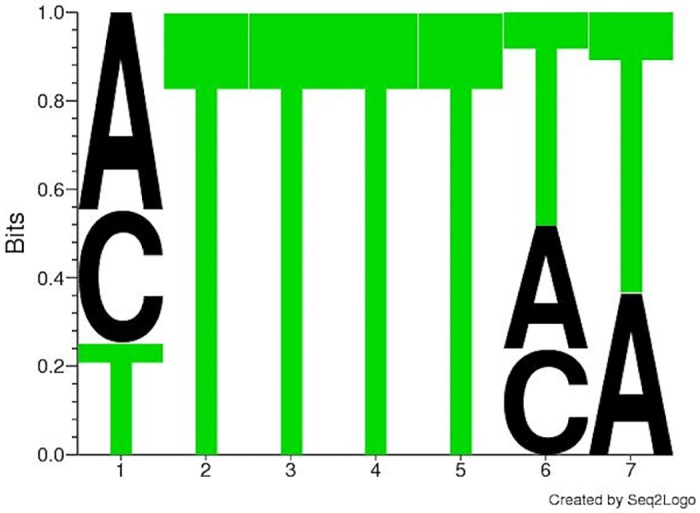
Position Frequency Matrix for the top motif identified by DREME for HuR.

We next sought to compare the *k*-spectrum kernel [24] results on HuR to the discriminative motif finder DREME [22]. The top motifs identified by DREME [22] were of length 7 (best E-value from motifs of length 7 to 13). The top DREME [22] motif, **HUUUUHW**, was found in 2,871 out of 3,642 sequences and had an E-value of 6.6e-143 (See Fig 2 for associated sequence logo.) The over-represented *k*-mers associated with this motif are listed in the Table 2. Both discriminative methods, the *k*-spectrum kernel method and DREME, discovered HuR motifs that were consistent with the published motifs, though the *k*-spectrum kernel method returned a longer motif.

**Fig 3:**
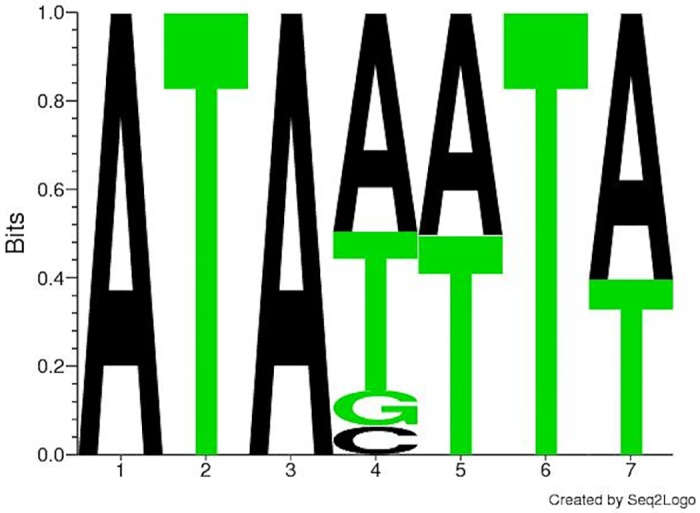
Position Frequency Matrix for the top motif identified by DREME for TTP.

We utilized a similar approach to validate the *k*-spectrum kernel on the prediction of TTP binding motifs. The *k*-spectrum kernel method was reasonably accurate, classifying the test data with a 88.8% accuracy rate. The top scoring features were AU-rich 9-mers, including the well known nonamer AUUUAUUUA (type 2 RRE) [31] that is specific to TTP binding targets [32]. DREME also identified AU-rich motifs, but shorter in length (7 nt) with the top DREME motif, **ATANWTW** scoring an E-value of 8.7e-743. This motif was found in 2,871 out of the 4,626 sequences. See Fig 3 for associated sequence logo. Table 3 compares the *k*-mers found by the *k*-spectrum kernel [24], DREME [22], and the published TTP motifs.

**Table 3:** Discriminative methods recover known K-mers for TTP. Top feature *k*-mers from the *k*-spectrum kernel and over-represented k-mers from DREME are compared to the published k-mers [4]. For the *k*-spectrum kernel, determined feature weights are provided.

Method	Success Rate	Sensitivity	PPV	ROC
*k*-spectrum	88.8	95.5	84.1	95.6
DREME	91.4	91.57	77.09	93.4
		**Published Motifs** [4]	***k*-spectrum Features and Weights**	**DREME over-represented *k*-mers**
		UUAUUUAUU	UUAUUUAUU, 6.44	AUAAAUA
		UUAUUUA	AUUUAUUUAUU, 5.4	
		UAUUUAUU	UAUUUAUUU, 5.34	AUAUUUA
		UUUA	UUUAUUUAU, 5.17	AUAUUUU
		AUUUA	AUUUAUUUA, 4.72	AUAUUUA

We next sought to determine whether we could improve on these models using feature engineering. Feature engineering is the process of transforming raw data into features that better represent the underlying problem to the predictive models, resulting in improved model accuracy on unseen data. As a great deal is already known about both HuR and TTP, we wondered if the addition of known features, namely that HuR binds U-rich and TTP binds to AU-rich regions, would improve the performance of our *k*-spectrum kernel models. The engineered features, normalized by sequence length, were:

- 4 features: Number of As, Us, Gs or Cs found in each sequence (aCount, uCount, gCount, cCount).
- 2 features: Number of contiguous As or Us found in each sequence (aRepeatedCount, uRepeatedCount).
- Total number of AU-rich dimers (AA, AU, UA, UU) found in each sequences (auCount).

The *k*-spectrum kernel method [24] was run using these additional features on both the HuR and TTP datasets and evaluated against the same parameters as the models developed without feature engineering. Table 4 lists the results of incorporating feature engineering into the *k*-spectrum kernel method.

**Table 4:** Feature engineering does not consistently improve model performance. Performance (success rate, sensitivity, PPV and ROC) for models for HuR and TTP incorporating engineered features.

RBP	Success Rate	Sensitivity	PPV	ROC
HuR	82.4	86.4	79.7	89.7
TTP	85.2	91.2	81.5	92.6

The engineered features topped the list for both proteins, suggesting that the generalized binding preferences are high quality descriptions of the binding site preferences of these RBPs. Interestingly, the top two features (uCount and auCount) were identical for both HuR and TTP, only in different orders, suggesting that both proteins indeed have similar binding preferences. The relative similarity of scores for these features on TTP further suggests that TTP binds to both AU-rich and U-rich sites somewhat equivalently. Table 5 enumerates the high scoring features for both HuR and TTP after the addition of engineered features. It is interesting to note, however, that inclusion of these engineered features did not consistently improve the performance of the model(See Table 4), indicating that the *k*-mers identified adequately recover the generalized feature descriptors.

**Table 5:** Engineered features obtain the top weights in the model. Top ten features along with their weights discovered by the *k*-spectrum kernel method when feature engineering is incorporated.

HuR (Feature, Weight)	TTP (Feature, Weight)
uCount, 1142.8	auCount, 1488.15
auCount, 956.9	uCount, 1462.98
uRepeated, 671.43	uRepeated, 894.11
UUUUUUUU, 46.69	aCount, 337.70
UUUAUUUU, 30.21	UUAUUUAUU, 38.17
AUUUUUUU, 19.88	UAUUUAUUU, 32.69
UUUUAUUU, 19.64	AAUAUUUAU, 26.33
UUUUUUUC, 17.99	AUAUUUAUU, 25.83
UUUUCUUU, 16.68	UUUAUUUAU, 20.89
UUAUUUUU, 15.63	AUUUAUUUA, 20.22

### Discrimination between HuR and TTP using k-spectrum models

Since both the proteins had similar high-scoring features we next sought to determine whether the models could discriminate between the binding sites of HuR and TTP. Hence, we sought to determine whether it could identify *k*-mers that distinguish between HuR and TTP binding. To this end, we utilized the three categories of peaks (HuR only, both HuR and TTP, and TTP only) in different scenario combinations (see Table 6) as both positive and negative sets. We reasoned that peaks bound only by HuR may harbor features distinct to its binding relative to TTP, and vice versa. For each case, we utilized both the *k*-spectrum kernel and DREME for analysis.

**Table 6:** Four different test scenarios to identify motifs shared and specific to each RBP.

Test	Positive	Negative
Scenario 1	Only HuR	Only TTP + (HuR And TTP)
Scenario 2	Only TTP	Only HuR + (HuR And TTP)
Scenario 3	(HuR And TTP)	Only HuR + Only TTP
Scenario 4	Only HuR	Only TTP

First, we sought to identify the features key to the HuR only dataset (the positive) distinct from TTP and common clusters (scenario 1 of Table 6). The resulting *k*-spectrum model identified U-rich features as the most discriminative. As shown in Table 7, it was able to discern between the two sets with a 79% balanced success rate. DREME identified over-represented *k*-mers were similar to those identified by the *k*-spectrum kernel, but typically shorter in length (Table 7), a pattern that was consistent across all four scenarios. Therefore, we focus our subsequent discussion on the *k*-spectrum results.

**Table 7:** Discriminative methods evaluate distinct subsets of the HuR and TTP dataset to identify both shared and specific sequence features. The performance of the *k*-spectrum kernel on distinct subsets of the data. Top eight *k*-mers from *k*-spectrum kernel (by weight) and DREME (by E-value). The test scenarios correspond to Table 6.

	Scenario 1	Scenario 2	Scenario 3	Scenario 4
	Only HuR vs (Only TTP and Common)	Only TTP vs (Only HuR and Common)	Common vs (HuR and TTP specific)	HuR-specific vs TTP-specific
Positive Sequences	3206	4161	467	3206
Negative Sequences	5667	4712	7367	4161
Balanced Success Rate	78.1	72.8	62.9	80.7
ROC	85.2	80.5	68.7	88.1
*k*-spectrum Features				
	UUUUUU	UAAUAUUUA	UUUUAUUUAA	UUUUGUUUU
	**UUUUUUU**	AAUAUUUAU	UUUAUUUAA	**UUUUUUUUUUU**
	UUUUUUUU	CUAUUUAUU	UUUAUUUA	**UUUUUUUUUC**
	**UUUUUUUUC**	**UAUUUAUUA**	**AUUUUUUAUU**	AUUUUUUUUU
	UUUUGUUUU	**UAUUUAUUUA**	UUUUAUAUU	UUUUUUUUUU
	UUUUUUUU	**AUUUAUUUAU**	UAUUUUUUUUA	**UUUUUUUU**
	**CUUUUUUUUU**	**UUAUUUAAU**	AUUUAUUUU	AUUUUUU
	**UUUUUUC**	AUAAAUAU	UAUUAUUUU	**CUUUUUU**
DREME Motifs				
	**UUUUUUU**	UAUUUAU	UUUUAUUU	**UUUUUUU**
	UUUUCUU	AUUUAUU	UAUUUAUAA	UUUUUGU
	**UCUUUUU**	AUUUAUA	UAUUUAUUC	AUUUUUU
	UCUUUUU	AUAAAUU	UAUUUAUUA	**CUUUUU**
	AUUUUUU	AUAUAUA	UAUUUAUAU	UUCUUUU
	AAUUUUU	AUUAAUA	UUUUUUUUUU	UUGUUUU
	CUCUUUU	UUUAUAA	**CUUUUUUUUU**	**CUCUUUU**
	AACUUUU	**AUUUUAUA**	UUUUUCUUUU	AACUUUU

To identify features specific to the TTP dataset, we employed TTP only as the positive and all of HuR and common clusters as the negative dataset (scenario 2 in Table 6). The discriminating features were AU-rich, with several containing one or more overlapping repeats of AUUUA. Further analysis of this repeating pentamer revealed that it was found 206 times in the TTP only sequences, only 25 times in the HuR only sequences, and 11 times in the common sequences suggesting that it is indeed a discriminating feature for TTP binding.

**Fig 4:**
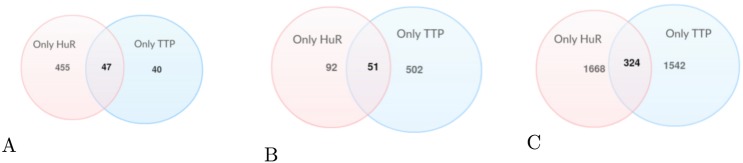
Analysis of Feature *k*-mers. (A) Number of occurrences of HuR specific feature *k*-mers in each dataset. (B) Number of occurrences of TTP specific *k*-mers in each dataset. (C) Number of occurrences of common *k*-mers in each dataset.

We next sought to identify the features that were typical of commonly bound regions (scenario 3 in Table 6). The model identified AU-rich features, though they had appreciable fewer A’s than those identified for TTP only. This suggests that *k*-mers intermediate between the U-rich features of HuR and the AU-rich features of TTP can be recognized by either protein. The success rate of the model, however, was discernibly lower (by 10–15 percentage points) than the HuR only and TTP only models. In line with the drop in accuracy, we noted that most instances of the top *k*-mers were found in the HuR only (1668 instances) and TTP only (1542 instances) datasets rather than the combined set (324 instances) (See Fig 4).

Finally, we wondered whether the inclusion of these common events was reducing the accuracy of our HuR specific and TTP specific models. To test this, we ran one last scenario, namely where the HuR only is used as a positive set and the TTP only as a negative (scenario 4 in Table 6). The resulting feature set qualitatively resembled those found for earlier HuR models, both the discriminative one (scenario 1 in Table 6) and the HuR independent model (Table 2), but the success rate improved a little relative to both models. While the increase in accuracy is small (2–3%), it does suggest that HuR binds a distinctly more U-rich motif than TTP and that discrimination by sequence features is possible.

### Discrimination between HuR and TTP using multi-task learning and domain adaptation technique

Given the limitations of a pure *k*-spectrum kernel approach at identifying features typical of commonly bound regions (results for scenario 3 in Table 6), we next employed the feature augmentation technique [28] in domain adaptation along with multi-task learning to identify not only the features specific to each domain but also those that are shared by the domains. We combined features with domain information to construct a combined model *M*_*B*_. We then compare this model to the domain specific models *M*_*H*_ (HuR) and *M*_*T*_ (TTP).

First, we conducted an out-of-domain test on *M*_*H*_ and *M*_*T*_ where the test data belonged to a different domain than the model. As seen in Table 8, the results show that the HuR model was able to discriminate TTP binding sites with a higher ROC score than the TTP model on HuR data. However, given our feature engineering results, it is not possible from this data alone to distinguish whether this indicates that the HuR dataset is simply more diverse (e.g. HuR binds a broader range of targets) or whether the TTP protein is actually more discriminative.

**Table 8:** Domain adaptation and multi-task learning identifies domain specific (prefaced with HuR or TTP) and shared (no prefix) *k*-mers. Performance metrics and top twenty *k*-mers are compared for the HuR, TTP and combined model (see main text for description of models).

Test Data/Model	HuR Model (*M*_*H*_)	TTP Model (*M*_*T*_)	Combined Model (*M*_*B*_)
HuR	89.4	87	89.4
TTP	91.2	95.6	94.5
Combined	NA	NA	92.5
Features			
	UUUUUUUUU	UAUUUAUUU	HuR
	UUUUAUUUU	UUAUUUAUU	**HuR_UUUUUUUUU**
	UUUUCUUUU	UUUAUUUAU	UUUUUUUUU
	UUUAUUUUU	AAUAUUUAU	UUUUAUUUU
	AUUUUUUUU	AUUAUUAUU	UAUUUAUUU
	UUUUUCUUU	AUAUUUAUU	UUAUUUAUU
	UUUUUAUUU	CUAUUUAUU	UUUAUUUUU
	UUUUUUUUC	AUUUAUUUA	UUUUUAUUU
	CUUUUUUUU	AUUAUUUAU	**HuR_UUUUAUUUU**
	UUUCUUUUU	UAUUUUUAU	AUUUUUUUU
	UUUUUUUCU	UAAUAUUUA	**TTP_UUAUUUAUU**
	UUUUUUUUA	UAUUAUUAU	**HuR_UUUAUUUUU**
	UUUUUUCUU	AUUUUUAUU	**HuR_AUUUUUUUU**
	UUAUUUUUU	UCUAUUUAU	**TTP_UAUUUAUUU**
	UUUUUUUAA	UUAUUAUUA	UUUUUUUUC
	UAUUUUUUU	UUAUUAUUU	UUAUUUUUU
	UUCUUUUUU	UUUAUUAUU	AUUUUAUUU
	AUUUUUAUU	UUUUAUUUA	**HuR_UUUUUUUUC**
	UUUUGUUUU	UUUUUAUUU	UUUUUUUUA
	UCUUUUUUU	AUUUAUUUU	CUUUUUUUU
	UUAUUUUUA	AUUUUAUUU	**TTP_AUUUAUUUA**

Then the combined model was run against each domain data as well as the combined dataset. The intuition behind the combined model is that it should predict HuR, TTP, and combined data with at least the same ROC score as the individual models since it contains the original set of features of both domains. As seen in the Table 8, the combined model *M*_*B*_ had the same ROC score for HuR data-set, and a slightly lower (less than 1%) ROC score for TTP data set. This suggests that the combined model may have a slight HuR bias, possibly stemming from the fact that HuR targets are typically longer in length. Consistent with this possibility, the combined model’s top features were HuR domain specific.

Interestingly, several of the domain specific features are also identified as shared. For example, HuR_UUUUUUUUU and UUUUUUUUU are identified as the second and third top *k*-mers. This indicates that poly-U tracks are recognized by both RBPs, hence it is a shared feature, but that it has higher significance in the HuR domain. In fact, all domain specific *k*-mers were also identified as shared except the last one, e.g. TTP_AUUUAUUUA, which corresponds to the known TTP specific nonamer. This is consistent with the feature identified by the TTP model (See Table 3). Despite the fact that only one *k*-mer (in the top twenty *k*-mers) is uniquely domains specific, the combined model ROC score was 92.5%, a slight improvement over the out-domain capabilities of the HuR only model in predicting TTP. This suggests that the combined model does identify distinguishing features for TTP that contribute to its success, despite the fact that they are not in the top ten features.

## Discussion

Two discriminative methods, the *k*-spectrum kernel method [24] and DREME [22], both discovered HuR and TTP motifs that were consistent with the published motifs; the HuR *k*-mers were predominantly U-rich, and AU-rich for TTP. While the success rate of these methods was comparable, the *k*-spectrum kernel method had higher sensitivity and PPV values than DREME. With discriminative methods, sensitivity and specificity are often trade-offs. This is likely the case here, as DREME had a slightly better success rate. In some cases, a higher sensitivity is preferred, particularly when subsequent experiments will validate the predicted sites and there is a cost to missing potential targets.

Additionally, relative to DREME [22], the *k*-spectrum kernel method identified slightly longer *k*-mers that provide additional insight into the flanking regions around the core motif. HuR is known to bind its targets using two RNA binding domains. Wang et. al. [33] found that HuR binding to *c-fos* RNA involved an 11-base segment 5′-AUUUUUAUUUU-3′, in support of a longer recognition sequence. Given the success of the *k*-spectrum kernel method at identifying binding motifs that matched known published motif of HuR and TTP, this method could be potentially used to identify *de novo* binding sites of other RBPs.

The case of HuR and TTP binding is particularly paradoxical, as both proteins recognize similar sequence features. In fact, feature engineering did not consistently improve the accuracy with which the *k*-spectrum kernel discriminated between RBP set and the control set. However, the engineered features did bubble up to be highly discriminative, suggesting that the binding sites of HuR and TTP are indeed simply U/AU-rich. Likewise, the features identified as domain specific using multi-task learning were often also shared features (such as HuR_UUUUUUUU and UUUUUUUU, HuR_UUUUAUUUU and UUUUAUUUU) suggesting that both proteins are capable of recognizing these *k*-mers but with distinct affinities. These results both confirm and provide further evidence for the hypothesis that these RBPs do indeed share similar binding preferences and varying affinity shifts decide which RBP would bind to a particular target.

Not all RNA binding proteins utilize primary sequence for recognition. Instead, some RNA binding proteins may recognize secondary structure. The co-variation inherent in conserved secondary structures is significantly more difficult to detect than primary sequence. It is unclear how well a *k*-spectrum kernel approach would fare when the target motif is structural. A careful examination of how well various discriminative methods perform when the target contains structural elements is a field of future research.

## Conclusion

To summarize, the discriminative methods were able to identify binding motifs of both RNA binding proteins. The *k*-spectrum kernel method provided additional insight of nucleotides around the binding sites. Neither feature engineering nor domain adaptation identified specific protein specific *k*-mers, further corroborating the extent to which these proteins recognize similar sequence features. Despite this, the increased *k*-mer length and sensitivity of the *k*-spectrum approach suggest this is an attractive approach for predicting unknown binding motifs of other RBPs.

## Supporting information

S1 Fig *k*-spectrum kernel method. (A) Example showing 3-mers of a given string AAUAAGUC. (B) Feature map showing all possible *k*-mers and the number of times a particular *k*-mer appears in the string. (C) Feature map for sequence AAUAAGUC that satisfies (3,1) condition for the 3-mer AAU.
(TIFF)

S1 Appendix Additional description of the *k*-spectrum kernel method including algorithm used to build the model. Summary of experimental methods and analysis on HuR and TTP datasets.
(PDF)
