# Correction: Discriminating between HuR and TTP binding sites using the *k*-spectrum kernel method

**DOI:** 10.1371/journal.pone.0175988

**Published:** 2017-04-12

**Authors:** Shweta Bhandare, Debra S. Goldberg, Robin Dowell

The following information is missing from the Funding section of the paper: Publication of this article was funded by the University of Colorado Boulder Libraries Open Access Fund.
