# Supplementary figures and images for: Discriminating between HuR and TTP binding sites using the k-spectrum kernel method
Source: PLoS One. 2017 Mar 23;12(3):e0174052. doi: 10.1371/journal.pone.0174052 (PMC5363848; doi:10.1371/journal.pone.0174052)

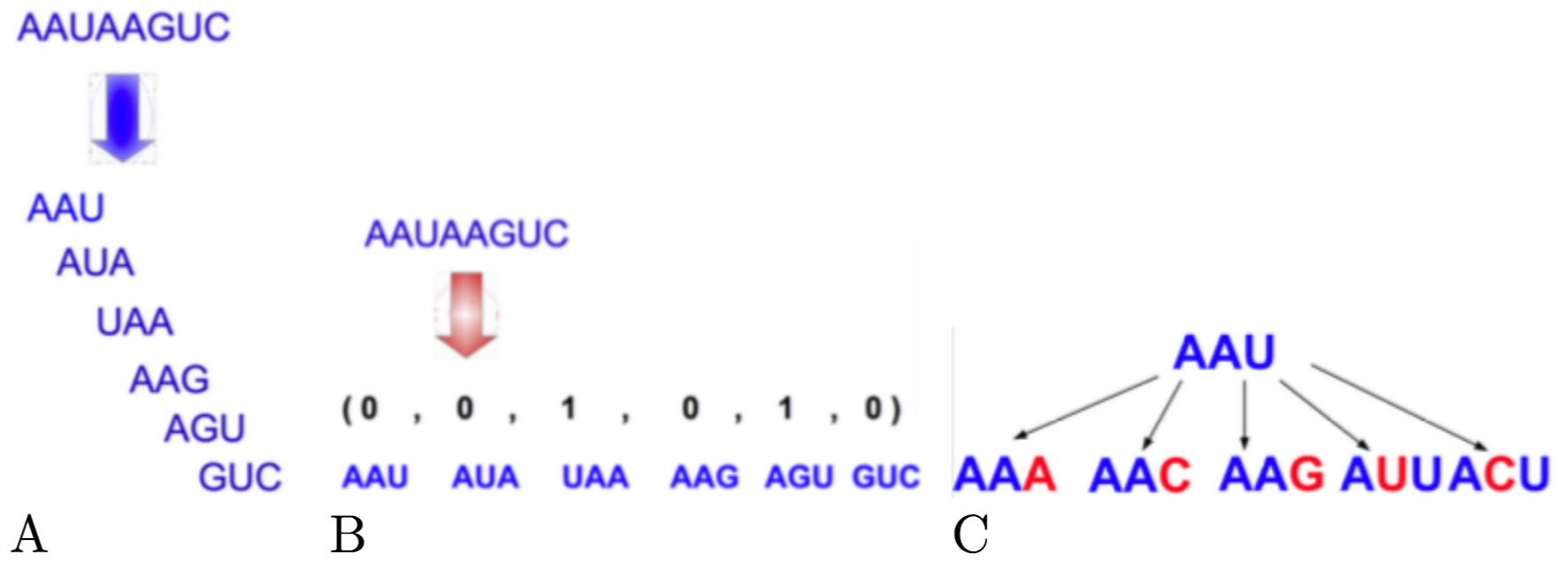

Supplement: S1 Fig — (A) Example showing 3-mers of a given string AAUAAGUC. (B) Feature map showing all possible k-mers and the number of times a particular k-mer appears in the string. (C) Feature map for sequence AAUAAGUC that satisfies (3,1) condition for the 3-mer AAU. (TIFF) [file pone.0174052.s001.tiff]
