# Supplementary material for: Discriminating between HuR and TTP binding sites using the k-spectrum kernel method
Source: PLoS One. 2017 Mar 23;12(3):e0174052. doi: 10.1371/journal.pone.0174052 (PMC5363848; doi:10.1371/journal.pone.0174052)
Supplement: S1 Appendix — Summary of experimental methods and analysis on HuR and TTP datasets. (PDF) [file pone.0174052.s002.pdf]

S1 Appendix

Shweta D. Bhandare, Debra S. Goldberg, Robin D. Dowell

Machine Learning Methods

Machine learning algorithms have been used to solve problems in bio-informatics. A basic learning model typically consists of a learning module, a decision-making module, a feedback module that communicates with the learning module about the performance of the algorithm, and a problem generator module that suggests actions that could lead to new or informative experiences. A discriminative approach is used where both positive and negative example sets are used for model training. Positive examples are known to belong to a particular class, and negative otherwise, and a learning algorithm is used to distinguish between the two classes.

A support vector machine (SVM) is a type of machine learning algorithm that finds a linear equation (hyperplane) that optimally separates two classes (sets of data) by maximizing the margin between the classes. The data points lying on the boundaries are called support vectors. When the sets are not linearly separable, a *kernel* function may be used that maps the data into a higher dimensional space where the sets are better able to be linearly separated. When used in conjunction with SVMs, kernel methods create very strong discriminative methods, and can be employed to classify mRNA sequences to predict if they bind to a specific RBP or not.

k-spectrum Kernel Method

The k-spectrum string kernel is a kernel function that works on strings (sequences). The kernel method breaks the sequences down into sub-strings that are of a fixed length k; these k-length sub-strings are known as k-mers. The intuition of this method is that the function measures the similarity between the sets of k-mers in sequences a and b; the more similar the k-mers are between sequences, the higher the value of a string kernel $K(a, b)$ will be.

The k-spectrum [1, 2] of a biological sequence is the set of k-mers it contains. The k-spectrum kernel visualizes this data in a feature map, which is indexed by all possible k-mers and gives the number of times each k-mer appears in the string (See Figure k-spectrum kernel method: k-mers, k-spectrum map, mismatches)

Fig S1: k-spectrum kernel method: k-mers, k-spectrum map, mismatches

Mismatches are very common in biological sequences, and to account for this Leslie et al. [1] proposed the mismatch string kernel where the feature map is modified to include mis-matches. The idea is to create a set of all k-length sequences β belonging to sequence A that differ by at most m mismatches. This set of sequences is also called the *mismatch neighborhood.* The feature map for sequence x will be the sum of all k-mers that satisfy the (k, m) condition. For example, the feature map under the $(3, 1)$ condition for the k-mer AAU is the set of all sequences of length 3 that differ from AAU by at most 1 mismatch.

The ability of SVMs to classify sequences into binary categories has applications in several facets of biological inquiry. The string kernel function [1] has also been used to determine homology of protein sequences that share a remote evolutionary relationship by Teramoto et al. [3].

Being able to define similarity features and map the sequences in the vector space makes kernel methods attractive especially when the training data is limited. Another advantage of using kernel functions is computational cost since the size of the kernel matrix depends only on the number of training examples and is independent of the dimension d the data is mapped into.

Algorithm to build k-spectrum kernel models

Listing 1: Algorithm to build k-spectrum kernel model

```
bestROC = -numpy.Inf
K1 = [8, 9, 10, 11, 12, 13]
K2 = [8, 9, 10, 11, 12, 13]
Cs = [ 10**x for x in xrange( -10, 5 ) ]
for k1 in K1:
    for k2 in [x for x in K2 if x >= k1]:
        for C in Cs:
          trainData = get_spectrum_data(trainSeqFasta, k1, k2)
          s = svm.SVM(C=C)
          s.train(trainData)
          results = s.stratifiedCV(trainData, numFolds=10);
          roc   = results.getROC();

          if roc > bestROC:
              trainData.save(dataFile);
              s.save(modelFile)
              bestROC = roc;
              bestC = C;
              bestK1 = k1;
              bestK2 = k2;
bestC, bestK1, bestK2 are obtained here.
```

HuR and TTP experimental methods

HuR dataset:

- Unstressed HeLA cell were used in three PAR-CLIP experiments
- 4SU in normal and 6SG in SILAC and normal medium
- Reads were aligned to the hg19 genome co-ordinates
- SAMTools [4] was used to generate pile-up file
- Custom scripts were run on the pileup output to identify clusters (continuous read coverage)
- Clusters were scored based on: a) Number of conversion events (T-C and G-A) and b) Number of overlapping reads with variable start and end positions in the same cluster were assigned higher quality score
- Within the clusters, the position with higher number of conversion events was computed which coincides with the preferred position of cross-linking
- 7-mers with less than ten occurrences were discarded and they compared the frequency of the 7-mers with the RefSeq [5] 3UTR sequences as background set

TTP dataset:

- Reads were aligned to the hg19 genome co-ordinates
- SAMTools [4] was used to generate pile-up file
- Reads that were less than 20 nt were discarded
- Used T-C and G-A number of these conversion events as quality measure
- Reads with T-to-C mismatches were gathered using PARalyzer [6] to retain only those that mapped to a single genomic location with the number of mis-matches as the criteria
- Computed the signal to noise ratio (SNR) by calculating the occurrence of known RNA recognition elements (RRE) in the TTP mRNA sites relative to the background set (3UTR sequences). The RREs were 40-250 times more enriched than the background set.
- The AUUUA pentamer was relatively more enriched in the TTP sites whereas the UUUUU and U-stretches flanked by C on either side were enriched in the HuR sites.

References

[1] Leslie C, Eskin E, Noble WSS. The Spectrum Kernel: A String kernel for SVM protein classification. Pacific Symposium on Biocomputing Pacific Symposium on Biocomputing. 2002; p. 564–575.

[2] Shawe-Taylor J, Cristianini N. Kernel Methods for Pattern Analysis. New York, NY, USA: Cambridge University Press; 2004.

[3] Teramoto R, Aoki M, Kimura T, Kanaoka M. Prediction of siRNA functionality using Generalized String Kernel and Support Vector Machine. FEBS Letters. 2005;579(13):2878–2882. doi:10.1016/j.febslet.2005.04.045.

[4] Li H, Handsaker B, Wysoker A, Fennell T, Ruan J, Homer N, et al. The Sequence Alignment/Map format and SAMtools. Bioinformatics. 2009;25(16):2078–2079.

[5] Pruitt K, Tatusova T, Brown G, Maglott D. NCBI Reference Sequences (RefSeq): current status, new features and genome annotation policy. Nucleic Acids Res. 2012;0(Database issue):D130–D135.

[6] Corcoran D, Georgiev S, Mukherjee N, Gottwein E, Skalsky R, Keene J, et al. PARalyzer: definition of RNA binding sites from PAR-CLIP short-read sequence data. Genome Biology. 2011;12(8):R79+. doi:10.1186/gb-2011-12-8-r79.
